# How Is the History of Early Traumatic Exposure Associated With the Psychopathological Outcomes of COVID-19 Related Lockdown and Subsequent Re-opening in People With Eating Disorders?

**DOI:** 10.3389/fpsyt.2021.789344

**Published:** 2021-12-08

**Authors:** Giammarco Cascino, Francesca Marciello, Giovanni Abbate-Daga, Matteo Balestrieri, Sara Bertelli, Bernardo Carpiniello, Giulio Corrivetti, Angela Favaro, Caterina Renna, Valdo Ricca, Pierandrea Salvo, Cristina Segura-Garcia, Patrizia Todisco, Umberto Volpe, Patrizia Zeppegno, Palmiero Monteleone, Alessio Maria Monteleone

**Affiliations:** ^1^Department of Medicine, Surgery and Dentistry “Scuola Medica Salernitana”, University of Salerno, Salerno, Italy; ^2^Department of Neuroscience, Eating Disorders Center for Treatment and Research, University of Turin, Turin, Italy; ^3^Unit of Psychiatry, DAME, University of Udine, Udine, Italy; ^4^Department of Mental Health, ASST Santi Paolo e Carlo, Milan, Italy; ^5^Section of Psychiatry, Department of Medical Sciences and Public Health, University of Cagliari, Cagliari, Italy; ^6^Department of Mental Health, Asl Salerno, Salerno, Italy; ^7^Department of Neuroscience, University of Padova, Padua, Italy; ^8^Mental Health Department, Center for the Treatment and Research on Eating Disorders, ASL Lecce, Lecce, Italy; ^9^Psychiatry Unit, Department of Neurological and Psychiatric Sciences, University of Florence, Florence, Italy; ^10^Eating Disorders Centre Portogruaro, AULSS 4 Veneto Orientale, San Donà di Piave, Italy; ^11^Department of Medical and Surgical Sciences, University Magna Graecia of Catanzaro, Catanzaro, Italy; ^12^Eating Disorders Unit, Casa di Cura “Villa Margherita”, Arcugnano, Italy; ^13^Section of Clinical Psychiatry, Department of Clinical Neurosciences/DIMSC, Università Politecnica delle Marche, Ancona, Italy; ^14^Department of Translational Medicine, Psychiatry Institute, Università del Piemonte Orientale, Novara, Italy; ^15^Department of Psychiatry, University of Campania L. Vanvitelli, Naples, Italy

**Keywords:** COVID-19, eating disorder, childhood maltreatment, stress, psychopathology

## Abstract

The negative impact of COVID-19 pandemic on people with Eating Disorders (EDs) has been documented. The aim of this study was to evaluate whether a history of traumatic experiences during childhood or adolescence was associated with a higher degree of psychopathological worsening during COVID-19 related lockdown and in the following re-opening period in this group of people. People with EDs undergoing a specialist ED treatment in different Italian services before the spreading of COVID-19 pandemic (*n* = 312) filled in an online survey to retrospectively evaluate ED specific and general psychopathology changes after COVID-19 quarantine. Based on the presence of self-reported traumatic experiences, the participants were split into three groups: patients with EDs and no traumatic experiences, patients with EDs and childhood traumatic experiences, patients with EDs and adolescent traumatic experiences. Both people with or without early traumatic experiences reported retrospectively a worsening of general and ED-specific psychopathology during the COVID 19-induced lockdown and in the following re-opening period. Compared to ED participants without early traumatic experiences, those with a self-reported history of early traumatic experiences reported heightened anxious and post-traumatic stress symptoms, ineffectiveness, body dissatisfaction, and purging behaviors. These differences were seen before COVID-19 related restrictions as well as during the lockdown period and after the easing of COVID-19 related restrictions. In line with the “maltreated ecophenotype” theory, these results may suggest a clinical vulnerability of maltreated people with EDs leading to a greater severity in both general and ED-specific symptomatology experienced during the exposure to the COVID-19 pandemic.

## Introduction

The outbreak of coronavirus disease 2019 (COVID-19) pandemic has led to a dramatic loss of human life worldwide and presents an unprecedented challenge to public health, food systems and the world of work. The economic and social disruption caused by the pandemic was devastating and led countries around the world to adopt social and physical distancing to contain virus transmission (1). Closures and restrictions worldwide promoted social isolation and loneliness (2–7). Increased exposition to the Internet and social media messages contributed to the fear of being infected (8–12). As a result of such a stressful situation, a clear-cut increased prevalence of anxiety and depression occurred in the general population (13–25), while people with a pre-existing psychiatric condition showed a heightened vulnerability in terms of physical and mental distress (26–31) as well as a higher frequency of most severe outcomes of the infection (32, 33), emphasizing the need to involve psychiatrists in the management of the emergency (34–41).

Among vulnerable psychiatric patients, people with eating disorders (EDs) have been reported to experience a worsening of their specific psychopathology, although variability in the degree of worsening has been observed across the studies (42–53). Furthermore, a marked impairment in general psychopathological symptoms has been observed in these patients, which tended to persist after the easing of COVID-19 related restrictions (54, 55). Several factors, such as social isolation, family conflict, disruption in routine activities, and everyday life, heightened exposure to ED-specific media messages and fear of contagion, may have contributed to the vulnerability of people with EDs to the impact of COVID-19 pandemic (50, 56).

Traumatic experiences during development have been acknowledged as important risk factors for several psychiatric disorders (57), including EDs (58). Clinical features (for instance, in terms of age at onset of the disorder and psychiatric comorbidity) as well as the identification of specific biological markers in patients with childhood maltreatment led Teicher and Samson (57) to suggest the existence across individuals suffering from a psychiatric condition of a “maltreated ecophenotype” subgroup with possibly different clinical presentation, biological underpinning, and prognosis. This perspective offers a functional understanding of symptoms highlighting their developmental influences (59).

In accordance with this hypothesis, people with EDs and a history of childhood maltreatment have been reported to show an earlier age at symptom onset, a greater symptom severity and a more frequent concomitance of other psychiatric conditions (60), a poorer treatment response (61), as well as a heightened biological and emotional vulnerability to acute social stress exposure (62) and specific gray and white matter alterations (63), highlighting the need of a multidisciplinary approach to their management (64).

As the COVID-19 pandemic encompasses different types of stressors, ranging from family and social to health and economic adversities, it may provide the opportunity to explore *in vivo* the effects of an acute traumatic event in people with a potential vulnerability to acute stressors (65–68). According to the maltreated ecophenotype hypothesis, an increased vulnerability to the pandemic distress may be hypothesized in people with EDs and a history of early maltreatment. Thus, the aim of this study was to evaluate whether a history of traumatic experiences during childhood or adolescence was associated with a higher degree of psychopathological worsening during COVID-19 related lockdown and in the following re-opening period.

We conducted a secondary analysis of data obtained through an online survey filled in by Italian participants with EDs after the easing of lockdown measures (54). We hypothesized that people with EDs and history of early traumatic experiences would exhibit greater worsening of general and ED-related psychopathology during the COVID-19 lockdown and the following re-opening compared to people without early exposure to adverse experiences.

## Materials and Methods

### Participants

Patients previously admitted to specialist ED units located in different regions of Italy and diagnosed with anorexia nervosa (AN), atypical AN, bulimia nervosa (BN), binge-eating disorder (BED), and other specified feeding or eating disorders (OSFED) according to the DSM-5 criteria were asked to fill in an anonymous online survey. The diagnosis was made by expert psychiatrists through face-to-face clinical interviews at admission. Participants had to meet the following inclusion criteria: (a) no comorbid schizophrenia or bipolar disorder; (b) no intellectual disability; (c) absence of physical comorbidity not related to the ED. Participants were invited to participate in the survey during a 20-day period from 1st June 2020 up to and including 21st June 2020. The local ethical committee communicated us that, in line with local legislation and national guidelines, the completion of an anonymous online survey did not require their approval. The consent was to be considered implicit when participants accepted to fill in the survey.

### Study Design and Measures

The online survey included a broad range of items aimed to assess the impact of the COVID-19 pandemic on the mental health of people with EDs.

Participants were asked to report their age, gender identity, geographic location (Italian Region), duration of illness, and undergoing treatments. Four different survey data collectors (one for each main ED diagnosis) were created. Each local investigator sent to the patients of her/his ED unit an email including a hyper-link to the survey from data collectors of the corresponding diagnosis and received completion feedback from patients.

The survey included a range of quantitative questions regarding both general and ED-specific psychopathology and it took ~20 min to be completed. Explored dimensions were anxiety, depression, post-traumatic stress symptoms (PTSS), obsessive-compulsive symptoms (OCS), panic symptoms, insomnia, suicide ideation. Questions related to these dimensions were adapted from the following questionnaires: Generalized Anxiety Disorder 7 (69), Patient Health Questionnaire 9 (70), PTSD Checklist for DSM-5 (71), and Obsessive-Compulsive Inventory (72). Items included were selected as the most consistent with the DSM-5 criteria for each psychiatric disorder. Questions specifically related to EDs psychopathology and behaviors included in the survey were adapted from the Eating Disorders Inventory [EDI-2; (73)]. The selected questions were representative of each EDI-2 subscale. Moreover, participants were asked to account for the use of laxatives and diuretics and the level of physical activity, which were reported as episode/week and hours/day, respectively.

Questions referred to three different time periods: 2 weeks before the spread of COVID-19 emergency; the lockdown period or “Phase 1” (that in Italy covered the months of March and April 2020); 2 weeks after the end of lockdown or “Phase 2” (that in Italy started on May 4, 2020). Each general psychopathology item was rated on a 10-point scale (0: not at all, 10: maximum), while ED specific items were rated on a 6-point scale, which was consistent with the original version of the EDI-2.

Participants were also asked if they had suffered any kind of traumatic experience such as sexual, physical, or emotional abuse or physical or emotional neglect (e.g., my family said hurtful things, I got hit badly enough to be noticed, I was sexually abused, I did not feel loved, I didn't have enough to eat), and if so if they had suffered it in childhood (up to the age of 10) or adolescence (age between 11 and 18 years). Based on the history of traumatic experiences, the participants were split into three groups: patients with EDs and no traumatic experiences, patients with EDs and childhood traumatic experiences, and patients with EDs and adolescent traumatic experiences.

### Statistical Analysis

Statistical analyses were performed using JASP software (2020). Analysis of variance (ANOVA) was performed to test significant differences among groups in demographic and clinical variables. ANOVA with repeated measures and self-report traumatic experience as between-subject factors and *post-hoc* Tukey's test were performed. Greenhouse-Geisser sphericity correction was applied where appropriate. Demographic variables that differed among groups were added as covariates in the analyses. A statistical threshold of *p* < 0.05 was set as significant.

## Results

### Clinical and Demographic Characteristics

Clinical and demographic characteristics of the whole sample and changes in general psychopathological dimensions over the three explored time periods are reported in Monteleone et al. (54).

Based on the self-reported traumatic experience, 91 participants were included in the group without traumatic experience (noMal), 119 participants in the group with a self-reported traumatic experience during childhood (childMal), and 102 participants in the group with a self-reported traumatic experience during adolescence (adoMal). The diagnostic composition of the sample was the following: 179 participants (57.4%) had a current AN or atypical AN, 63 (20.2%) had a current BN, 48 (15.4%) had a current BED, 22 (7.05%) had a current OSFED. No statistically significant difference emerged in the diagnostic composition of the three groups (χ^2^ = 5.29, *p* = 0.51). The groups differed in age and self-reported illness duration [*F*_(2,309)_ = 3.38, *p* = 0.03 and F_(2,309)_ = 4.12, *p* = 0.02, respectively], but not in body mass index (BMI) and treatment duration [*F*_(2,309)_ = 0.09, *p* = 0.91 and *F*_(2,309)_ = 2.19, *p* = 0.11, respectively] (Table 1). *Post-hoc* Tukey's test revealed that noMal participants had an older age than adoMal participants (*t* = 2.58, *p* = 0.03) while childMal participants had a longer illness duration than adoMal participants (*t* = 2.85, *p* = 0.01). Therefore, age and treatment duration were entered as covariates in the ANOVA with repeated measures.

**Table 1:** Demographic characteristics of participants according to self-reported traumatic experiences.

	**No trauma (*n* = 91)**	**Childhood trauma (*n* = 119)**	**Adolescence trauma (*n* = 102)**	***F*_(2,308)_**	***p***
Age	31.71 ± 13.65	28.91 ± 11.97	27.26 ± 10.21	3.38	0.03
Body mass index	20.69 ± 7.48	20.98 ± 9.19	21.22 ± 8.27	0.09	0.91
Illness duration	8.69 ± 9.69	10.73 ± 10.71	7.17 ± 6.73	4.12	0.02
Treatment duration	4.85 ± 7.32	3.94 ± 4.81	3.23 ± 3.65	2.19	0.11
Anorexia nervosa	61 (34%)	64 (36%)	54 (30%)		
Bulimia nervosa	14 (22%)	27 (43%)	22 (35%)		
Binge eating disorder	11 (23%)	20 (42%)	17 (35%)		
OSFED	5 (23%)	8 (36%)	9 (41%)		

*OSFED, Other specified feeding and eating disorders*.

### General Psychopathology

**Figure 1:**
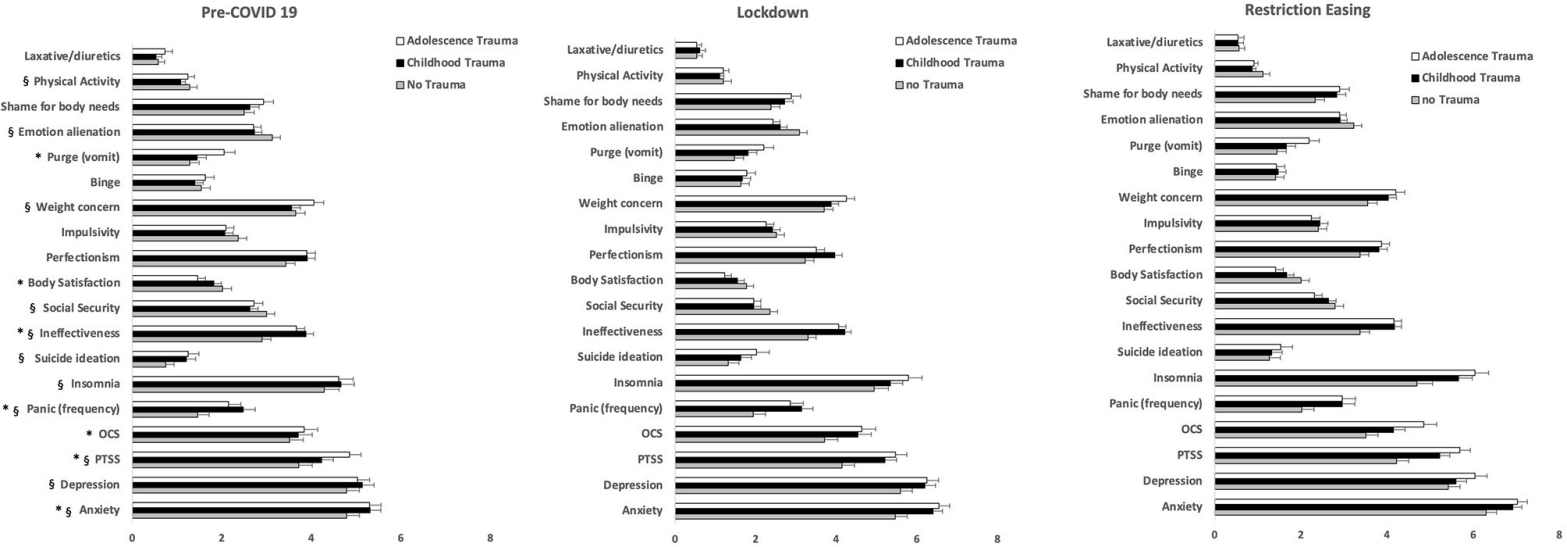
Scores of self-reported general and specific psychopathology and symptoms in participants with eating disorders according to self-reported history of early traumatic experiences. OCS, obsessive-compulsive symptoms; PTSS, post-traumatic stress symptoms. *Group effect: *p* < 0.05; ^§^time effect: *p* < 0.05.

Two way ANOVA with repeated measures showed a significant effect of time for symptoms of anxiety [*F*_(2,614)_ = 12.27, *p* = 5.93 × 10^−6^], depression [*F*_(2,614)_ = 4.19, *p* = 0.01], PTSS [*F*_(1.95,599.16)_ = 6.16, *p* = 0.002], panic [*F*_(2,614)_ = 3.41, *p* = 0.03], insomnia [*F*_(2,614)_ = 3.72, *p* = 0.02], suicide ideation [*F*_(1.87, 573.74)_ = 6.08, *p* = 0.003], but not for OCS [*F*_(1.57, 480.1)_ = 1.13, *p* = 0.31]. Indeed, compared to the pre-pandemic phase all but OCS scores were significantly higher after the start of COVID-19 related restrictions (Figure 1).

Between-group comparisons and *post-hoc* Tukey's test are summarized in Table 2.

**Table 2:** Between-group comparison of general and eating disorder related psychopathology scores.

	***F*_(2,307)_**	***p***	**Tukey *post-hoc***
Anxiety	3.81	0.02	noMal < childMal, adoMal
Depression	1.25	0.29	
PTSS	7.7	5.46 × 10^−4^	noMal < childMal, adoMal
OCS	3.18	0.04	noMal < adoMal
Panic (frequency)	4.07	0.02	noMal < childMal
Insomnia	2.61	0.08	
Suicide ideation	1.01	0.37	
Ineffectiveness	8.05	0.001	noMal < childMal, adoMal
Social security	1.29	0.28	
Body satisfaction	3.83	0.02	adoMal < noMal
Perfectionism	1.92	0.14	
Impulsivity	0.61	0.54	
Weight concern	2.21	0.11	
Binge	0.61	0.55	
Purge (vomit)	3.21	0.04	noMal < adoMal
Emotion alienation	1.47	0.23	
Shame for body needs	1.57	0.21	
Physical Activity	0.52	0.59	
Laxative/diuretics	0.06	0.94	

*PTSS, Post-traumatic stress symptoms; OCS, obsessive-compulsive symptoms; noMal, patients without history of trauma; childMal, patients with self-reported childhood trauma; adoMal, patients with self-reported adolescence trauma*.

*Post-hoc* Tukey's tests showed that anxiety and PTSS scores were significantly lower in noMal participants compared to both childMal (*t* = 2.54, *p* = 0.03 for anxiety and *t* = 2.44, *p* = 0.03 for PTSS) and adoMal groups (*t* = 2.29, *p* = 0.04 for anxiety and *t* = 3.91, *p* < 0.01 for PTSS), while OCS were significantly lower in noMal participants than in adoMal group (*t* = 2.52, *p* = 0.03) and panic was significantly lower in noMal participants than in childMal ones (*t* = 2.73, *p* = 0.02).

No significant time X group interaction emerged from the analyses. Participants' age and illness duration did not affect significantly these results with the exception for a marginal effect of age on OCS [*F*_(1,307)_ = 4.51, *p* = 0.04].

### ED-Related Psychopathology

Two way ANOVA with repeated measures showed a significant effect of time for ineffectiveness [*F*_(2,614)_ = 7.33, *p* = 0.001], social security [*F*_(2,614)_ = 9.57, *p* = 0.001], weight concern [*F*_(1.8,569.15)_ = 5.55, *p* = 0.005], emotional alienation [*F*_(2,614)_ = 8.35, *p* = 0.001], and physical activity [*F*_(1.9,588.45)_ = 5.23, *p* = 0.006], but not for body satisfaction [*F*_(1.76, 541.93)_ = 1.12, *p* = 0.32], perfectionism [*F*_(2,614)_ = 0.25, *p* = 0.77], impulsivity [*F*_(2,614)_ = 1.26, *p* = 0.28], binge [*F*_(2,614)_ = 2.70, *p* = 0.07], purge-vomit [*F*_(2,614)_ = 2.19, *p* = 0.11], shame of body needs [*F*_(1.89,580.5)_ = 0.71, *p* = 0.49], and laxative/diuretics abuse [*F*_(1.7,521.4)_ = 0.36, *p* = 0.66], demonstrating that, compared to the pre-pandemic period, these symptoms were significantly more severe after the beginning of COVID-19 related restrictions (Figure 1). Between-group comparisons and *post-hoc* Tukey's test are summarized in Table 2. *Post-hoc* Tukey's tests showed that ineffectiveness was lower in noMal participants compared to both childMal (*t* = 3.67, *p* < 0.01) and adoMal groups (*t* = 3.37, *p* < 0.01), while body satisfaction was lower in adoMal group than in noMal group (*t* = 2.69, *p* = 0.02) and purge-vomit was lower in noMal group than in adoMal group (*t* = 2.69, *p* = 0.04).

No significant time X group interaction emerged from the analyses. Participants' age and illness duration did not affect significantly these results except for the effects of age on binge [*F*_(1,307)_ = 12.15, *p* = 0.001], emotional alienation [*F*_(1,307)_ = 13.69, *p* = 0.001] and laxative/diuretics abuse [*F*_(1,307)_ = 5.00, *p* = 0.02] and the effect of illness duration on emotional alienation [*F*_(1,307)_ = 10.15, *p* = 0.002].

## Discussion

The present study investigated changes in general and specific psychopathology during the COVID-19 related restrictions and in the following re-opening period in a large population of Italian patients with EDs with or without a history of early traumatic experiences.

Compared to the pre-pandemic phase, both people with or without exposure to early traumatic experiences reported a worsening of general and ED-specific psychopathology during the COVID 19-induced lockdown and in the following re-opening period. Perceived social isolation, forced cohabitation and heightened family conflict, disruption in routine activities and everyday life, heightened exposure to social media messages, and fear of contagion, may have contributed to the vulnerability of people with EDs to the impact of COVID-19 pandemic (50, 56). In line with our study hypothesis, participants with EDs and a history of early traumatic experiences showed higher scores in general and ED-related psychopathology in the pre-pandemic period, during the pandemic-induced lockdown and in the following re-opening period compared to participants with EDs without early traumatic experiences. To our knowledge, only one previous study (51) investigated the effect of childhood maltreatment on the psychopathology course during the COVID-19 related lockdown. The authors found that childhood trauma significantly predicted COVID-19-related post-traumatic symptomatology in patients with AN. In line with these data, we observed significantly higher PTS scores in our population of participants with mixed ED diagnoses and history of early traumatic experiences. Furthermore, compared to participants with EDs without trauma history, people with EDs and history of childhood or adolescent traumatic experiences reported higher levels of anxiety, panic, and OCS also in the pre-pandemic period. These data are in line with research findings demonstrating that a greater symptoms severity characterize people with EDs and history of early traumatic experiences (60) and support the existence of a “maltreated ecophenotype” clinically different from non-maltreated patients (57).

The present data were collected during a traumatic event which included many kinds of stressors such as social isolation, impairment in the family, and/or individual's economic condition and fear of being infected. Therefore, the increased severity of anxious symptoms observed in maltreated patients with EDs is also consistent with recent experimental research (62), which hypothesized a biological and emotional vulnerability to stressful events in this subgroup of people with EDs. Previous studies identified an altered emotion regulation and cognitive bias toward social negative stimuli in people with EDs and early adverse experiences (74, 75). Thus, heightened rejection sensitivity and emotion regulation difficulties promoted by early traumatic experiences may contribute to explain the association of these experiences with a more severe psychopathology. This is also in line with the research evidence supporting the central role of affect regulation for mental health (76).

Surprisingly, people with EDs and a history of early traumatic experiences did not differ in depression, insomnia, or suicidal ideation from those without childhood adverse experiences. This may point to an increased vulnerability of maltreated people with EDs to anxiety symptoms rather than to depressive ones and/or may indicate that the COVID-19 pandemic may exacerbate one type of symptoms more than others, although these hypotheses remain speculative. However, depressive symptoms have been found to be worsened during the lockdown in all people with EDs, suggesting the need for clinicians to address them with *ad hoc* interventions (77).

As for ED-specific psychopathology, participants with early traumatic experiences reported heightened levels of ineffectiveness, body dissatisfaction, and purging behavior (namely, self-induced vomiting). These results are in line with previous research data. Indeed, low self-esteem and ineffectiveness have been proposed as possible mediators between childhood maltreatment and ED-specific symptoms (78–82). In addition, some studies (83, 84) have reported that a more severe purging behavior may be promoted by the need to regulate trauma-induced negative emotions by engaging in rash actions and risky behaviors. Finally, an association between childhood maltreatment and body dissatisfaction in adulthood has been reported in both community samples (85) and people with EDs (66, 86).

Other ED specific psychopathological variables (social security, perfectionism, impulsivity, weight concern, binge eating, shame of body needs, physical activity, and laxative/diuretics abuse) did not differ between participants with and without history of early maltreatment. In a previous analysis (54), these psychopathological variables worsened during the COVID-19 lockdown but, differently from general psychopatholgical symptoms, they returned to pre-pandemic levels in the re-opening period. This could be related to the fact that the easing of COVID-19 related restrictions could not mean real changes in the everyday life conditions, even if the survey was taken during June 2020 when the daily number of COVID-19 cases has dropped significantly in Italy. In the light of the high pre-pandemic symptom levels in people with EDs, we suggest that the observed lack of differences in those ED-related psychopathological variables between maltreated and non-maltreated people with EDs may be explained by a sort of ceiling effect, which hides a higher vulnerability in maltreated people with ED. Since for patients with EDs and history of early adverse experiences social situations could be stressful (62), this hypothesis can also be aligned with the observed lack of difference in the reactivity (e.g., time × group effect) of maltreated people with EDs to the pandemic restrictions as well as to the easing of COVID-19 related restrictions: the higher pre-pandemic levels of psychopathology seen in maltreated people with EDs may persist, hiding a different reactivity.

Remarkably, the age at the trauma occurrence (e.g., childhood or adolescence) did not contribute to the psychopathological differences observed in maltreated people with EDs. Previous studies have suggested that the time of the trauma occurrence may promote different biological sequelae (57), although no data have confirmed this hypothesis from a clinical perspective in EDs. However, the retrospective nature of our assessment may affect this finding, suggesting that further research is needed to explore this issue.

From a clinical perspective, these findings confirm the need to systematically assess the presence of adverse traumatic experiences in patients with EDs and to therapeutically address the ability to adapt to and face adversities (87, 88), likely impaired in this subgroup of patients with EDs. This may promote more personalized psychotherapeutic approaches (89–93), possibly allowing to overcome treatment resistance (94, 95). The widespread implementation of Internet-delivered psychological treatments (96–99), associated with the relatively positive attitudes toward e-therapies in people with eating problems (100), may be a way to increase access to psychological treatment for people with EDs. Finally, the important role of general psychopathological symptoms in maltreated people with EDs may support the transdiagnostic perspective recently proposed (101, 102) for the treatment of various emotional disorders.

Some limitations of the present study need to be acknowledged. First, the retrospective design of our assessment does not allow to exclude recall biases. Indeed, early traumatic experiences were retrospective and self-reported: however, this is the most common methodology of early trauma data collection in EDs (103). Moreover, the survey was proposed and retrieved clinicians who were following the participants with EDs, which might lead to a bias on reporting traumatic experiences. Regarding psychopathology assessment, the short duration of time that elapsed between the beginning of COVID-19 outbreak and data collection partially reduced this bias. Second, in line with the transdiagnostic ED perspective (104, 105) and with experimentally shown lack of differences among different ED diagnoses in the symptom response to stress exposure (62), we did not account for possible differences between the main (AN, BN, BED and OSFED) ED diagnoses. This area needs to be addressed by future studies. Moreover, the methodology of data collection may have affected the study findings, and the use of a new online survey may also underpin aspects of subjectivity. However, since we built the survey by adopting questions from validated psychometric questionnaires, we are confident that this limitation had a slight impact on our findings, although we cannot completely rule out this bias. The relatively small sample size, in particular the low number of participants with ED diagnosis other than AN, and the self-reported illness duration may affect the generalizability of our results. Finally, the cross-sectional design does not allow to make causal interpretations.

## Conclusions

People with EDs and history of early traumatic experiences reported heightened anxious and PTS symptoms, ineffectiveness, body dissatisfaction, and purging behaviors than those without early adverse experiences, regardless of the age when trauma occurred. These differences were seen before COVID-19 related restrictions as well as during the lockdown period and after the easing of COVID-19 related restrictions. In line with the “maltreated ecophenotype” theory, it is possible to hypothesize a vulnerability of maltreated people with EDs, leading to a greater severity in both general and ED-specific symptomatology experienced during the exposure to the stressful lockdown related to the COVID-19 pandemic. The persistence of this detrimental effect in the following re-opening period may suggest either a slower recover or a heightened sensitivity to the restoring social exposure in this patient subgroup.

## Data Availability Statement

The raw data supporting the conclusions of this article will be made available by the authors, without undue reservation.

## Ethics Statement

Ethical review and approval was not required for the study on human participants in accordance with the local legislation and institutional requirements. Written informed consent from the participants' legal guardian/next of kin was not required to participate in this study in accordance with the national legislation and the institutional requirements.

## Author Contributions

AM, FM, GCa, and PM designed the study, wrote the protocol, and wrote the manuscript. GA-D, MB, SB, BC, GCo, AF, CR, VR, PS, CS-G, PT, UV, and PZ collected data. GCa did statistical analyses. All authors contributed to the article and approved the submitted version.

## Conflict of Interest

The authors declare that the research was conducted in the absence of any commercial or financial relationships that could be construed as a potential conflict of interest.

## Publisher's Note

All claims expressed in this article are solely those of the authors and do not necessarily represent those of their affiliated organizations, or those of the publisher, the editors and the reviewers. Any product that may be evaluated in this article, or claim that may be made by its manufacturer, is not guaranteed or endorsed by the publisher.
